# Effect of some local plant extracts on fatty acid composition of fish (*Alestes baremoze*) during smoking and sun drying in the Far‐North region of Cameroon

**DOI:** 10.1002/fsn3.3636

**Published:** 2023-08-16

**Authors:** Goldefroy Tabanty Zambou, Noël Tenyang, Lucie Birault, Alice Kermarrec, Agnes Gacel, Germain Kansci, Anne Meynier, Sylvain Guyot, Roger Ponka

**Affiliations:** ^1^ Department of Biological Sciences, Faculty of Science University of Maroua Maroua Cameroon; ^2^ INRAE, UR BIA Nantes France; ^3^ INRAE, UR1268 BIA (Biopolymers, Interactions & Assemblies), PRP Group (Polyphenols, Reactivity, Processes) Le Rheu France; ^4^ Department of Biochemistry, Faculty of Science University of Yaoundé I Yaoundé Cameroon; ^5^ Department of Agriculture, Livestock and Derivated Products, National Advanced School of Engineering of Maroua University of Maroua Maroua Cameroon

**Keywords:** *Alestes baremoze*, *Allium cepa* leaves, antioxidants, fatty acids, *Moringa oleifera* leaves, smoking and sun drying, *Xylopia aethiopica* fruits

## Abstract

The objective of this study was to assess the antioxidant activities of three plant extracts (*Moringa oleifera* leaves, *Xylopia aethiopica* fruits, and *Allium cepa* leaves) and to evaluate their effects on the preservation of fish polyunsaturated fatty acids (PUFAs) during smoking and sun‐drying processes. PUFAs are highly prone to oxidation during fish processing. The plant extracts were analyzed for their polyphenol contents and were evaluated for their total antiradical capacity. The polyphenol components of each plant were characterized. The hydroethanolic and aqueous extracts were added to the fish at concentrations of 3, 6, 9, and 12 g/L and 10, 20, 30, and 40 g/L, respectively. Butylated hydroxytoluene (BHT) was used as a positive control at a concentration of 2 g/L to compare the antioxidant effects of the plant extracts. The treated fish was subjected to smoking or sun drying and the fatty acid composition of the fish lipid extract was assessed. The results showed that the total polyphenolic, flavonoid, and tannin contents varied significantly from one plant extract to the other (*p* < .05). The radical scavenging and FRAP increased significantly with the concentration of the plant extracts (*p* < .05). An HPLC analysis of the extracts led to the preliminary identification of four hydroxycinnamic acids in *M. oleifera* and *X. aethiopica*, one anthocyanin and one flavone glycoside in *M. oleifera*, and four flavan‐3‐ols in *X. aethiopica*. Moreover, eight flavonols were preliminarily identified in the three plants. Compared to the control product, these plant extracts significantly protected fish PUFAs from oxidation (*p* < .05). The aqueous extract of *A. cepa* at 40 g/L better preserved omega‐3 in fish during smoking and sun drying than the control product. Incorporating the three plant extracts during smoking and sun‐drying processes can effectively preserve the PUFAs in fish. Therefore, these plants are viable sources of natural antioxidants in the preservation of fish products.

## Graphical abstract

The total polyphenolic, flavonoid, and tannin contents varied significantly from one plant extract to the other. Compared to the control product, these plant extracts significantly protected fish PUFAs from oxidation. The aqueous extract of *A. cepa* at 40 g/L better preserved omega‐3 in fish during smoking and sun drying than the control product.

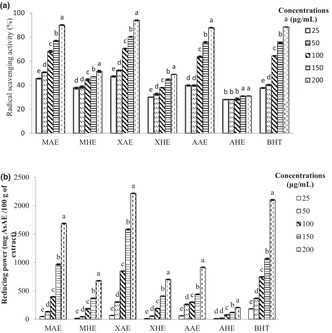

## 1 INTRODUCTION

Fish has been widely used as an excellent source of animal protein and other nutrients. It is a food rich in proteins of high nutritional value, lipids, minerals, and vitamins (Hantoush et al., 2014). In Cameroon, the national average consumption of fish was estimated at about 18.4 kg/inhabitant/year (FAO, 2017). Eating fish can prevent consumers from various diseases such as high blood pressure, coronary heart disease, cancer, and inflammatory disease since fish provide omega‐3 polyunsaturated fatty acids (PUFAs), eicosapentaenoic acid (EPA), docosahexaenoic (DHA) acids, and amino acids (Morales et al., 2015).

However, fish is one of the most fragile and perishable marine products after capture (Brigitte et al., 2005). The impediment in its preservation comes from its high moisture content (75%–80%) which constitutes a favorable environment for the development of bacteria. In developing countries, including Cameroon, smoking and sun drying are choice processes used to limit the deterioration of fresh fish and increase its shelf life (Kumolu‐Johnson et al., 2010). However, during smoking and sun‐drying processes of fish samples, the high temperatures and oxygen exposure lead to the oxidation of lipids content. Lipid oxidation can lead to changes in organoleptic properties, color, texture, appearance, and the release of undesirable flavors and odors (Kazuhisa, 2001). Extensive oxidation can also lead to a decrease in the nutritional properties of foods through the loss of components such as PUFAs (Cuvelier & Maillard, 2012). Furthermore, these oxidative reactions can potentially produce toxic compounds through the release of free radicals and reactive oxygen molecules that are harmful to human health and are implicated in degenerative conditions such as cancer, cardiovascular diseases, and early aging (Krishnaiah et al., 2010).

In order to delay lipid oxidation, synthetic antioxidants such as butylated hydroxytoluene (BHT), butylated hydroxyanisole (BHA), and tert‐butyl hydroquinone (TBHQ) have been used to maintain the quality and extend the shelf life of oils. However, their use is increasingly being contested and has even been banned in certain countries due to their potential health risks which include cancer and cardiovascular diseases (Krishnaiah et al., 2010). Even so, many consumers have negative perception of the effect of synthetic antioxidant. In addition, BHA and BHT are quite volatile and easily decompose at high temperatures (Thorat et al., 2013). In order to overcome these challenges, food industries are searching for alternative antioxidants that are more stable and from natural sources, which in general, are supposed to be safer.

Many studies have been reported on natural sources of antioxidants. The most common sources of phenolic compounds currently exploited for use in foods are rosemary (Chammem et al., 2015), berries (Aladedunye & Matthäus, 2014), coffee (Budryn et al., 2011), tea leaves (Kmiecik et al., 2018), and olive leaves (Zribi et al., 2013). To expand these studies, other natural sources of antioxidants are required. According to literature, the antioxidant potential of natural plant extracts is mainly related to their content in phenolic compounds. *M. oleifera*, which grows naturally in many countries, is a powerful natural antioxidant because of its high content in flavonoids, tocopherols, vitamin C, and other phenolic compounds (Pakade et al., 2013). *A. cepa* is a common vegetable that is widely consumed all over the world. It is also a powerful natural antioxidant because it contains good amounts of flavonoids which are the largest group of phenolic compounds, along with quercetin (Archivio et al., 2007; Tiwari & Cummins, 2013). *X. aethiopica* also has a high level of phenolic compounds, flavonoids, and tannins; and demonstrates good antioxidant activity (Sokamte et al., 2019).

In the Far North Region of Cameroon, *M. oleifera*, *X. aethiopica*, and *A. cepa* grow in abundance and their leaves and fruits are highly consumed by the local populations. Their richness in polyphenols makes them good candidates for the evaluation of their antioxidant capacity during fish processing. The objective of this study was to investigate the effect of aqueous and hydroethanolic extracts of *M*. oleifera leaves, *X. aethiopica* fruits, and *A*. *cepa* leaves on the oxidative stability of processed fish by evaluating its fatty acid composition.

## 2 MATERIALS AND METHODS

### 2.1 Materials

#### 2.1.1 Chemicals

Standard antioxidants (gallic acid, quercetin, catechin, and BHT), 95° ethanol, 95° methanol, the Folin–Ciocalteu reagent, sodium carbonate (Na_2_CO_3_), 2,2‐diphenyl‐1‐picrylhydrazyl (DPPH), aluminum chloride (AlCl_3_), sodium acetate, vanillin, 35% hydrochloric acid (HCl), 99% acetic acid, phosphate buffer, potassium hexacyanoferrate (K_3_Fe(CN)_6_), trichloroacetic acid (TCA), ferric trichloride (FeCl_3_), ascorbic acid, chloroform, and anhydrous sodium were obtained from Sigma‐Aldrich. Standard fatty acid methyl esters (C_4_–C_22_) and boron trifluoride‐methanol (14% BF_3_/CH_3_OH, v/v) were purchased from Sigma‐Aldrich. Internal standard of the colon (C17:0) was purchased from Sigma‐Aldrich. *n*‐Hexane was from Biosolve Chimie SARL. Acetic acid, HPLC grade methanol, and acetonitrile were obtained from Carlo Erba Reagents. Formic acid and Sodium fluoride were purchased from VWR Prolabo. Standards of epicatechin, hyperoside, and ideain were purchased from Extrasynthese. All the chemicals were of analytical grade.

#### 2.1.2 Laboratory materials

Filter paper (Whatman No.4, England), rotary evaporator (Stuart Bibby Scientific, RE300DB), electric air‐dried oven (Memmert UN30, Zirndorf), UV–vis spectrophotometer (PerkinElmer, Lamda 950, UV/VIS, UK), blender (Panasonic), electronic analytical balance (Ohaus), freezer (Innova, IN200), electronic hot plate (VWR, Cole‐Parmer, Thermo Scientific, France), gas chromatography system (GC Clarus 690, Perkin Elemer), and reverse‐phase Purospher STAR Hibar HR RP18 end‐capped column (150 mm × 2.1 mm, 3 μm, thermostated at 30°C, Supelco) in a LC system that is composed of: a solvent degasser (SCM1000, Thermo Scientific), a binary high‐pressure pump (1100 series, Agilent Technologies), a Surveyor autosampler thermostated at 4°C (Thermo Scientific), equipped with a UV–visible photodiode array detector (UV6000 LP, Thermo Scientific) and an ion trap mass spectrometer with electrospray ionization source (LCQ Deca, Thermo Scientific).

#### 2.1.3 Plant materials

The *X. aethiopica* fruits, and *M. oleifera* and *A. cepa* leaves used in this study were bought from the Maroua local market in March 2020. This town is situated in the Far North Region of Cameroon and is located between Latitudes 10° and 13° North and Longitudes 13° and 16° East (RGPH, 2010). The samples were transported in sealed bags to the Food Biochemistry Laboratory of the University of Maroua. The *A. cepa* leaves were washed with tap water, left to drain, and cut into small pieces. These cut leaves, the *X. aethiopica* fruits, and the fresh *M. oleifera* leaves were washed and dried at 50°C for 48 h in an electric air‐dried oven (Memmert UN30, Zirndorf). The dried samples were ground using a blender (Panasonic) to obtain fine powders that can pass through a 0.5 mm sieve. The powders were then used for the preparation of the aqueous and hydroethanolic extracts.

#### 2.1.4 Animal material

Adult fish (*A. baremoze*) was bought from fishermen on the shores of Lake Maga (Far North Region of Cameroon) in April 2021. It was transported immediately in iceboxes to the Food Biochemistry Laboratory of the University of Maroua. After washing to remove external dirt and cleaning with disposable paper towels, the fish was eviscerated, washed anew with distilled water, and left to drain. The weight varied from 900 to 1000 g and the sizes were between 40 and 50 cm.

### 2.2 Methods

**FIGURE 1:**
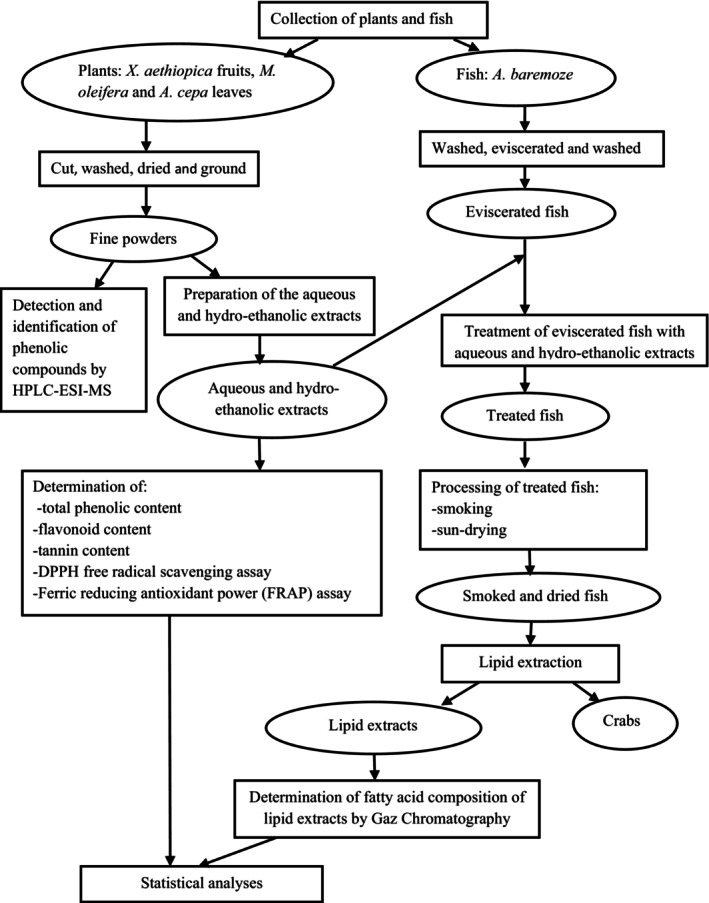
Flow diagram showing methodology followed in the experimentation.

Figure 1 presents flow diagram showing methodology followed in the experimentation and the analysis performed at each stage.

#### 2.2.1 Extraction of plant antioxidants

The extraction was performed according to the method described by Friedman et al. (2006). Twenty grams (20 g) of each plant powder were extracted with 500 mL of hydroethanolic mixture (40/60: v/v) for 48 h at room temperature (RT, ~24°C). The mixture was consistently shaken during the process and was strained through Whatman N° 4 filter paper. The residue was extracted again with 250 mL of the same solution to ensure maximum recovery of phenolic compounds. The combined extracts were subjected to rotary evaporator at 40°C under reduced pressure to remove the solvent. The residue was obtained by drying the extract in an oven at 45°C until it became solid and of constant weight.

On the other hand, 20 g of each plant powder was dissolved in 500 mL of distilled water. The mixtures were heated to boiling point, submitted to reflux for 15 min, and were filtered through clean Whatman Paper N° 4 while hot. The filtrates (aqueous extracts) were cooled to RT, and then dried in an oven at 45°C until they became solid and of constant weight. They were then stored at 4°C prior to further analysis.

#### 2.2.2 Phytochemical characterization of the extracts

##### Determination of total phenolic content

The total phenolic content of the extracts was evaluated using the Folin–Ciocalteu colorimetric method as described by Gao et al. (2000). In a 5 mL test tube, 20 μL of a 2 g/L extract (water or aqueous ethanol) was added, followed by the Folin–Ciocalteu reagent (0.2 mL) and distilled water (2 mL). After incubating the mixture for 3 min at RT, 1 mL of 20% sodium carbonate solution was added and it was re‐incubated for 2 h under the same conditions. The absorbance (Abs) of the resulting blue‐colored solution was read at 765 nm with a spectrophotometer. The total phenolic content of the extract was calculated from the gallic acid (0–125 μg/mL) standard curve and was expressed in grams of gallic acid equivalents per 100 g of plant extract (g GAE/100 g of dry extract).

##### Determination of flavonoid content

The flavonoid content of the different samples was determined by the method of Mimica‐Dukic´ (1992). Essentially, 0.1 g of each plant extract was homogenized with 10 mL of methanol. To 0.1 mL of the mixture diluted to a tenth was added 1 mL of aluminum chloride reagent (133 mg of crystalline aluminum chloride and 400 mg of crystalline sodium acetate dissolved in 100 mL of methanol). After homogenization, two drops of acetic acid were added. The mixture was homogenized again and Abs was read at 430 nm using a spectrophotometer. The quantity of flavonoids was calculated from the calibration curve of quercetin standard solutions (0–250 μg/mL) and expressed in grams of quercetin equivalents per 100 g of plant extract (g QUE/100 g of dry extract).

##### Determination of tannin content

Tannin levels in the plant extracts were determined by the method of Bainbridge et al. (1996). According to it, 0.1 g of each plant extract was homogenized with 10 mL of methanol. To 0.2 mL of the mixture diluted to a tenth were added 2 mL of reactive reagent (50 g of vanillin and 4 mL of hydrochloric acid in 100 mL of distilled water). The mixture was incubated at 30°C for 5 min and Abs was recorded at 500 nm with a spectrophotometer. The quantity of tannins was calculated from the calibration curve of catechin standard solutions (0–50 μg/mL) and expressed in grams of catechin equivalents per 100 g of plant extract (g CAE/100 g of dry extract).

##### Determination of antioxidant activities

###### DPPH free radical scavenging assay

The radical scavenging ability of the plant extracts was determined according to the method of Popovici et al. (2009). A total of 4.5 mL of a 0.002% (w/v) alcoholic solution of DPPH was added to 0.5 mL of different concentrations (125, 250, 500, 1000, and 2000 μg/mL) of samples and standard solutions in order to have final concentrations of 25–200 μg/mL. BHT, a synthetic antioxidant, was used as the positive control. The mixtures were kept at RT in the dark for 30 min, after which the Abs of the samples, control, and blank were measured at 517 nm in comparison with methanol. The antiradical activity (AA) was determined using the following formula:

$$\mathrm{AA}\;\left(\%\right)=\left[\left({\mathrm{Abs}}_{\text{control}}-{\mathrm{Abs}}_{\text{sample}}\right)\times 100/{\mathrm{Abs}}_{\text{control}}\right].$$

###### Ferric reducing antioxidant power (FRAP) assay

The antioxidant potential of the plant extracts was also evaluated by their ability to reduce iron (III) to iron (II) according to the method of Oyaizu (1986). Aliquots of 0.5 mL of plant extracts at various concentrations (125, 250, 500, 1000, and 2000 μg/mL) were individually added to 1 mL of phosphate buffer (0.2 M, pH 6.6) and 1 mL of 1% (w/v) aqueous potassium hexacyanoferrate solution, well shaken, and incubated at 50°C for 30 min. After incubation, 1 mL of 10% (w/v) TCA solution was added to stop the reaction, and the mixture was centrifuged at 3000 rpm for 10 min. A total of 1.5 mL of supernatant, 1.5 mL of distilled water, and 0.1 mL of 0.1% (w/v) ferric trichloride solution were mixed and incubated for 10 min, and Abs was read at 700 nm on a spectrophotometer. Once more, BHT was used as the positive control. The reducing antioxidant power was calculated from the calibration curve of ascorbic acid (0–125 μg/mL) standard solutions, and expressed in milligrams of ascorbic acid equivalents per 100 g of plant extract (mg AsAE/100 g of dry extract) by using the following formula:

$$\text{FRAP}\;\left(\mathrm{mg}\;\text{AsAE}/100\;g\;\text{of}\;\mathrm{dry}\;\text{extract}\right)=\left[\mathrm{Abs}\times \mathrm{Fd}\times V\times 100/\left(\mathrm{Tp}\times a\right)\right];$$

where Fd = dilution factor, *V* = total extraction volume; Tp = test portion; *a* = slope of the calibration curve.

##### High‐Performance liquid chromatography (HPLC) and electrospray Ionization‐Mass spectrometry (ESI‐MS) analysis

The detection and identification of phenolic compounds in the three plant extracts were performed by HPLC paired with ESI‐MS. To that end, 50 mg of dried material was dissolved in 1200 μL of methanol containing 1% of acetic acid, followed by sonication for 15 min (Brasson 2200, USA). The procedure was repeated three times and the combined extracts were filtered through an injection flask (Mini uniprep Whatman 0.45 μm) for HPLC and injected into an HPLC‐UV–visible‐DAD‐ESI‐MS system. The analytic device was composed of an SCM1000 degasification system (Thermo Scientific), an autosampler (Model Surveyor, Thermo Scientific), an 1100 series binary pump (Agilent Technologies), and a diode array UV–visible detector (DAD, UV6000 LP, Thermo Scientific). The mass spectrometer (MS) was an ion trap (LCQ Deca, Thermo Scientific) equipped with an ESI source. A sample volume of 2 μL was injected into a Purospher STAR Hibar HR RP18 column (150 mm × 2.1 mm, 3 μm, thermostated at 30°C, Supelco). The mobile phase consisted of Solvent A (aqueous solution of 0.1% formic acid, v/v) and Solvent B (acetonitrile containing 0.1% formic acid, v/v). The following linear gradient elution was applied at a constant flow rate of 0.2 mL/min: initial, 3% B; 0–3 min, 7% B, linear; 3–21 min, 13% B, linear; 21–27 min, 13% B, linear; 27–40 min, 30% B, linear; 40–51 min, 50% B, linear; 51–53 min, 90% B, linear; 53–56 min, 90% B, linear; 56–58 min, 3% B, linear; 58–72 min, 3% B, linear; followed by washing and reconditioning of the column.

The UV–visible (UV–Vis) detection was performed in the 240–600 nm range. The ESI source was used in negative mode. The MS detection was carried out with the following parameters: MS spectra were acquired in full‐scan negative ionization mode in the m/z 50–2000 range to obtain the signals corresponding to the deprotonated [M‐H]^−^ molecular ions. The method also included the MS/MS‐dependent scan mode which was used to obtain the product ion spectrum of the main molecular ions detected on the chromatogram in the full‐scan mode. The collision energy was optimized at 35% (arbitrary units) to clearly observe the production of both parent and main daughter ions. Data were collected and processed by XCalibur Software (Version 1.2, Thermo Finnigan).

By comparison with available standards, the retention times, UV–Vis spectra, full MS spectra, and MS/MS spectra were used for complete identification. When the standard was not available, the criteria were used for partial identification only. Quantifications were carried out by integration of the peaks on UV–Vis chromatograms at 280 nm for flavanols, 320 nm for hydroxycinnamic acids, 350 nm for flavonols, and 520 nm for anthocyanins.

(−)‐Epicatechin, 5‐caffeoylquinic acid, and procyanidin dimer B2 were quantified according to their own calibration curves, whereas other compounds were quantified “as equivalents” according to a reference compound belonging to the same polyphenol class and presenting a comparable UV–Vis spectrum. Thus, procyanidin oligomers were quantified in epicatechin equivalents, flavonols in hyperoside equivalents, and anthocyanins in ideain equivalents.

#### 2.2.3 Preparation of plant extracts and treatment of fish

##### Preparation of plant extracts

The concentrations used were chosen according to Foffe et al. (2020) who proposed values and average yields of plant extracts. The average yields (~30%) and powder weights (10, 20, 30, and 40 g) were exploited to assess the mass (3, 6, 9, and 12 g) of crude concentrated hydroethanolic extracts. These crude extracts and BHT were dissolved each in 1 mL of ethanol and then in 1 L of distilled water to give concentrations of 3, 6, 9, and 12 g/L for the extracts and 0.2 g/L for the BHT. This synthetic antioxidant (BHT) was used at the legal limit of 0.2 g/L (Duh & Yen, 1997). Besides, 10, 20, 30, and 40 g of each plant powder were also dissolved in 1 L of distilled water. The mixtures were then brought to boil and submitted to reflux for 15 min, after which they were filtered through clean Whatman Paper N° 4 while hot. The filtrates were then cooled to RT (Tenyang et al., 2020).

##### Fish treatment

The fish samples were treated and coded as follows: Control: Fish without extract and BHT (with water only); F: Fish; BHT: butylated hydroxytoluene; F + BHT0.2 g/L: Fish treated with butylated hydroxytoluene at concentration 0.2 g/L; F + MHE: Fish treated with *M. oleifera* hydroethanolic extracts; F + MAE: Fish treated with *M. oleifera* aqueous extracts; F + XHE: Fish treated with *X. aethiopica* hydroethanolic extracts; F + XAE: Fish treated with *X. aethiopica* aqueous extracts; F + AHE: Fish treated with *A. cepa* hydroethanolic extracts; F + AAE: Fish treated with *A. cepa* aqueous extracts. The hydroethanolic extracts were used at concentrations of 3, 6, 9, and 12 g/L while the aqueous extracts were used at concentrations of 10, 20, 30, and 40 g/L. The treated fish samples were divided into two parts and each part was subjected to smoking and sun‐drying process.

#### 2.2.4 Processing of fish

##### Smoking

One batch of fish was treated to smoking using the previously mentioned experimental design. The samples were spread out on smoking trays which were then stacked on a smoking 0.9‐m‐high oven fired with hard wood and marked at temperatures greater than 70°C. The process lasted for 12 h during which the samples were turned at intervals to ensure homogeneous drying. The dried smoked fish samples were packaged and sealed in bags that were stored at 4°C for further analyses.

##### Sun‐drying

The treated fish was cut into two equal halves along its longitudinal body axis from mouth to tail but left attached at the tail region. The pieces were then spread out on a traditional dryer braided with twigs of wood, exposed to open air, and protected by a mosquito net to prevent invasion by insects and other pests. They were subjected during the day (8 a.m.–5 p.m.) to ambient sunlight at temperatures between 25 and 40°C. The chunks were turned over from time to time to ensure homogeneous drying. The sun‐drying process took 3 days due to the climatic conditions during the drying period, the moisture content of the air was comparatively low. The dried pieces were packaged and sealed in bags that were stored at 4°C for further analyses.

#### 2.2.5 Lipid extraction

Lipids were extracted from the raw and processed fish according to the Bligh and Dyer (1959) method. One hundred grams (100 g) of the commodity was placed in a blender (Panasonic) to which 100 mL of chloroform and 200 mL of methanol were added and blended for 3 min. This was followed by the addition of 100 mL of chloroform and 100 mL of distilled water. The mixture was blended again for 1 min and then filtered. The final extraction was ensured by the addition of more chloroform to attain a proportion of 2:2:1.8 of chloroform, methanol, and distilled water, respectively. After separating the different phases in a funnel, the organic phase was collected and dried using anhydrous sodium. The organic solvent was then eliminated using a rotary evaporator at 45°C under reduced pressure. The lipid extracts obtained were put in dark glass bottles and stored at −20°C for further analyses.

#### 2.2.6 Determination of fatty acid composition of lipid extracts

The fatty acid composition of the lipids extracted from the smoked and sun‐dried fish was determined after transmethylation according to the method described by Morrison and Smith (1964). The analysis of the fatty acid methyl esters (FAME) was performed on a gas chromatograph (Clarus 690 GC, Perkin Elmer) paired with a splitless injector and a flame ionization detector. FAME were separated on a capillary column (DB 225, 30 m × 0.32 mm, film thickness 0.25 μm, Chromoptic) with H_2_ as the carrier gas set at a constant flow of 2 mL/min. The chromatographic conditions applied were as previously described by Fogang et al. (2017). Individual fatty acids were identified by a comparison of their retention times with those of a standard mixture. Results were expressed in percentage of each fatty acid (FA) in relation to the total identified fatty acids (TFA – g/100 g TFA).

#### 2.2.7 Statistical analyses

Data were presented as mean ± standard deviation (SD). Values were statistically analyzed by the one‐way analysis of variance (ANOVA) test using the IBM SPSS Statistics Version 20.0.1. Software Package. Differences were considered significant at *p* < .05 using the Duncan multiple‐range test. The principal component analysis (PCA) was carried out to gain an overview of the relationships among the experimental data. The correlation between the experimental parameters was evaluated by Pearson's correlation test at *p* < .05.

## 3 RESULTS AND DISCUSSION

### 3.1 Total phenolic, flavonoid, and tannin contents of extracts

**FIGURE 2:**
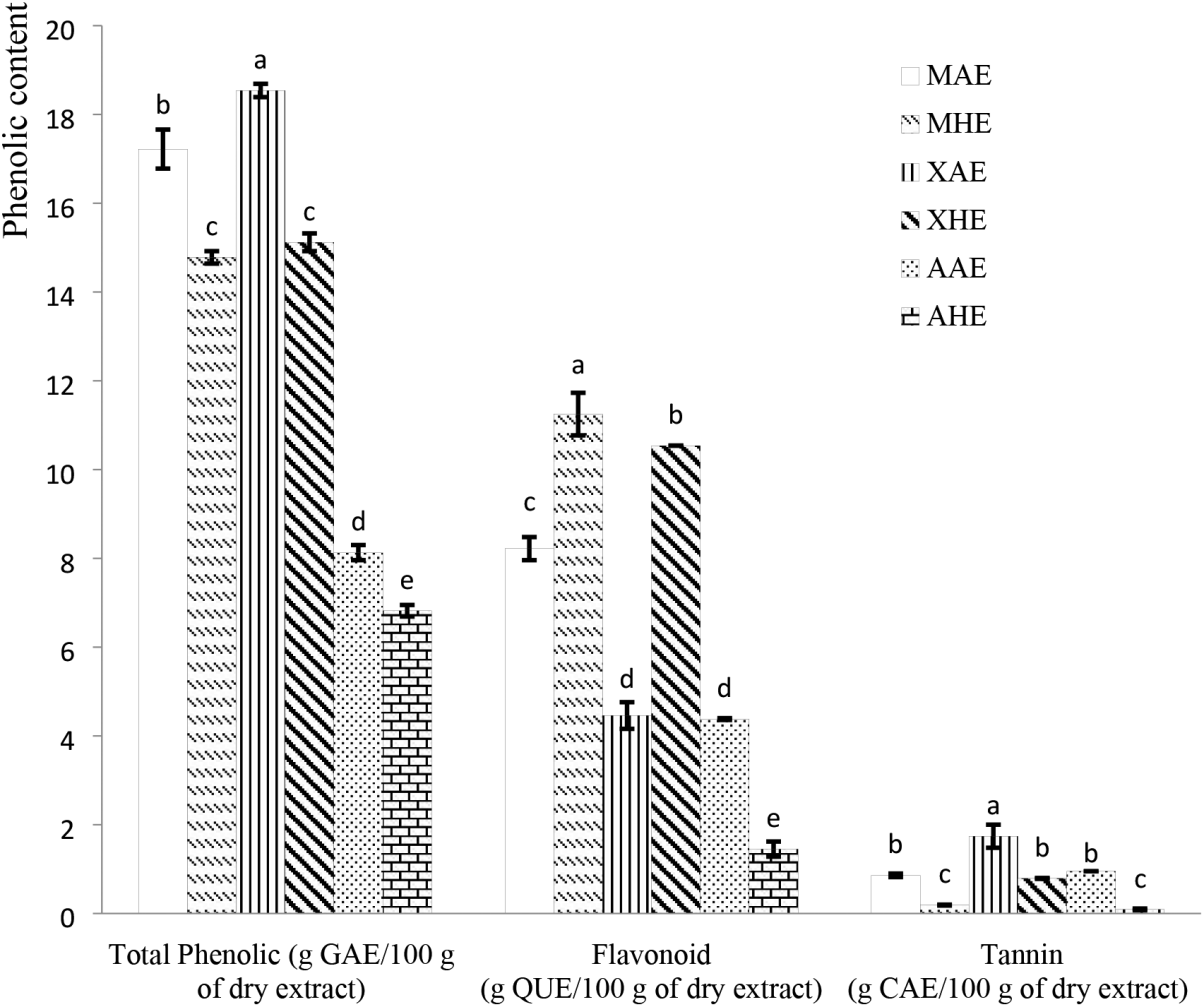
Total Phenolic, flavonoid, and tannin contents of plant extracts. AAE, *A. cepa* aqueous extracts; AHE, *A. cepa* Hydroethanolic Extracts; CAE, Cathechine Equivalent; GAE, Gallic Acid Equivalent; MAE, *M. oleifera* aqueous extracts; MHE, *M. oleifera* hydroethanolic extracts; QUE, Quercetin Equivalent; XAE, *X. aethiopica* aqueous extracts; XHE, *X. aethiopica* hydroethanolic extracts. Mean values for the same phenolic compounds with different superscript letters are significantly different (*p* < .05).

Figure 2 shows the total phenolic, flavonoid, and tannin contents of the different plant extracts. The total phenolic content was found to vary significantly from one plant extract to the other (*p* < .05). The values ranged between 6.8 and 18.5 g GAE/100 g of dry extract, with XAE presenting the highest level (18.5 g GAE/100 g of dry extract). For the same plant, the aqueous extracts (AE) had higher values than the hydroethanolic extracts (HE). The total phenolic levels found were similar to those obtained by Sokamte et al. (2019) in some selected spices from Cameroon (7–20 g GAE/100 g of dry extract). However, the values obtained in this study were higher compared to those obtained by Mendoza‐Taco et al. (2022) in *Moringa oleifera* extracts with 100% distilled water, 50% absolute ethanol, 50% distilled water, and 100% absolute ethanol (2.43, 1.10, and 2.61 g/100 g, respectively). The difference in the total phenolic content may be due to the state of physiological maturity of the plant and the solvent used (Du Toit et al., 2020; Oso & Oladiji, 2018).

Flavonoids constitute one of the most important phenolic groups in plants. Their contents varied significantly in the different plant extracts (*p* < .05), with values ranging from 1.4 to 11.2 g QUE/100 g of dry extract. The highest level was found in the *M. oleifera* hydroethanolic extracts (11.25 g QUE/100 g of dry extract). The values obtained were higher than those determined by Alikwe and Omotosho (2013) and Oso et al. (2018) on the same extract (4.90 g/100 g and 0.15 g/100 g) from Nigeria.

Condensed tannins are a group of phenolic compounds that result from the polymerization of flavanol units (Abdou et al., 2012). Their contents showed a significant variation among the plant extracts (*p* < .05), ranging from 0.09 to 1.74 g CAE/100 g of dry extract. The XAE presented the highest quantities of condensed tannins (1.74 g CAE/100 g of dry extract), although the registered values were lower than those reported by Sokamte et al. (2019) from Cameroon (14.57 g GAE/100 g). But, the values were higher than those observed by Hossain et al. (2020) (0.01 g GAE/100 g DM) in *M. oleifera* leaves methanolic extract. The difference noted may be due to the analytical techniques used, as well as the environment of the plant, the type of plant, the maturity, and/or the type of soil (Matinez‐Ramos et al., 2020; Mykhailenko et al., 2020).

### 3.2 Antioxidant activity of plant extracts

#### 3.2.1 DPPH test

**FIGURE 3:**
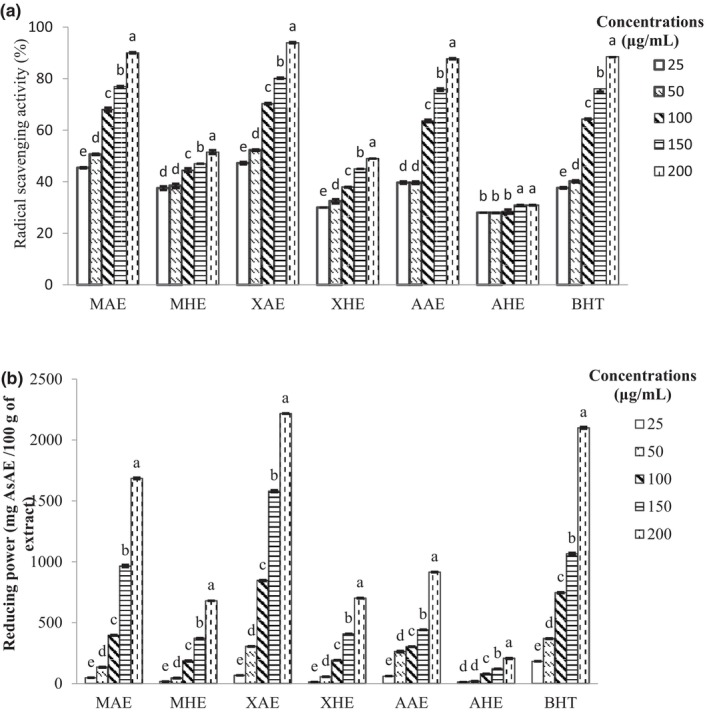
Antioxidant activity of plant extracts. (a) DPPH radical scavenging activity, and (b) Ferric reducing antioxidant power. AAE, *A. cepa* aqueous extracts; AHE, *A. cepa* hydroethanolic extracts; AsAE, ascorbic acid equivalent; MAE, *M. oleifera* aqueous extracts; MHE, *M. oleifera* hydroethanolic extracts; XAE, *X. aethiopica* aqueous extracts; XHE, *X. aethiopica* hydroethanolic extracts. Mean values in the same extract with different superscript letters are significantly different (*p* < .05).

The measurement of the ability of a molecule or substance to scavenge free radicals has become routine in testing the antioxidant property of plant extracts and is in fact their primary signature. The ability of an antioxidant to stabilize these radicals by donating its hydrogen is related to its potential capability to inhibit lipid oxidation (Matsubara et al., 1991). The DPPH radical has the capacity to extract labile hydrogen atoms. The ability of the plant extracts to scavenge the DPPH radical in comparison to BHT is presented in Figure 3a. It was observed that for all the solutions, this activity significantly increased with concentration (*p* < .05), and the XAE always had the highest values. It was therefore clear that the aqueous extracts, like BHT, are powerful free radical scavengers. Mendoza‐Taco et al. (2022), Oguntona et al. (2022), and Óscar et al. (2020) also demonstrated that *M. oleifera*, *X. aethiopica*, and *A. cepa* are powerful free radical scavengers. This observed effect could be due to their high phenolic content which, in many studies, has been reported to be related to antioxidant activity through this mechanism of action (Yin et al., 2019). It is also generally believed that the positions and total number of hydroxyl groups present in the aromatic constituents of the extracts offer better antioxidative properties (Parcheta et al., 2021).

#### 3.2.2 FRAP assay

The efficacy of a molecule or substance to reduce Fe^3+^ into Fe^2+^ by donation of its electron is also known as a good indicator of its antioxidant activity. The reaction leads to the formation of a Pearl's Prussian blue color which absorbs light at 700 nm (Seladji et al., 2014). Figure 3b shows the FRAP of the plant extracts compared to BHT. All the values were found to significantly increase with concentration (*p* < .05), and at 200 μg/mL, XAE exhibited the highest activity (2216.88 mg AsAE/100 g). These observations indicated that the plant extracts were also powerful ferric reducers. The FRAP might be attributed to the presence of phenolic compounds, mainly flavonoids, that have been proven to be powerful metal reducers (Zhou & Tang, 2017). The polyphenolic compounds in the extracts appeared to function as good electron‐ and hydrogen‐atom donors and therefore served to terminate radical chain reactions by converting free radicals to more stable products. The registered values were in correlation with those found by Sokamte et al. (2019) in the *X. aethiopica* extract from Cameroon (21,182 mg AsAE/100 g). Oso and Oladiji (2018) also demonstrated that the *X. aethiopica* extract is a powerful ferric reducer (1307.66 mg AsAE/100 g).

### 3.3 Elucidation of phenolic profiles of plant extracts by HPLC‐DAD‐ESI‐MS

**FIGURE 4:**
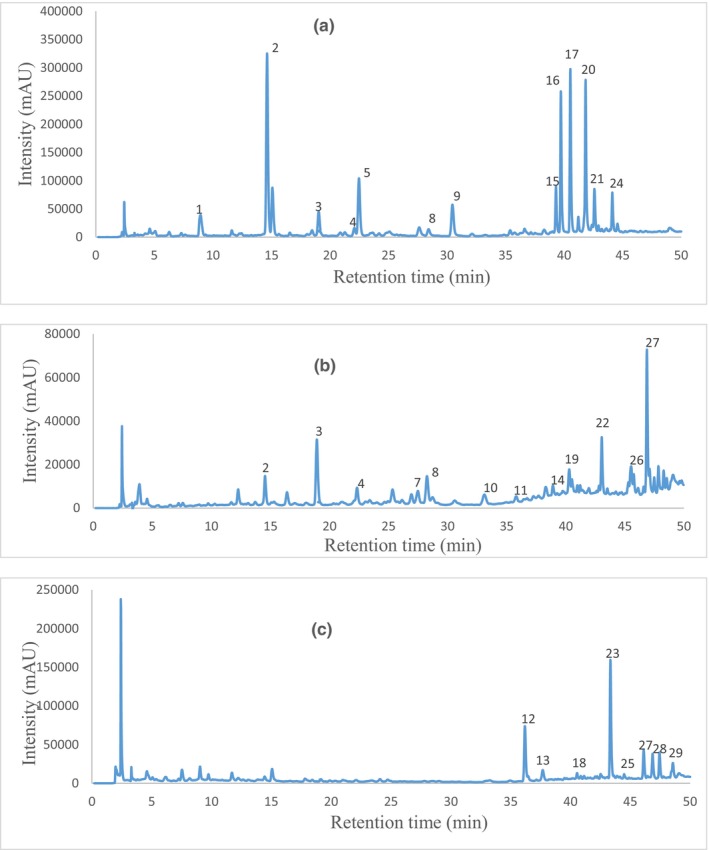
Reversed‐Phase UV–Visible HPLC chromatograms of methanolic extracts of *M. oleifera* leaves (a), *X. aethiopica* fruits (b), and *A. cepa* leaves (c) at 280, 320, 350, and 520 nm. Peak numbers correspond to (1) Unknown 1; (2) Caffeoylquinic acid isomer 1 (Hydroxy cinnamic acid); (3) *p*‐Coumaroylquinic acid isomer 1 (Hydroxy cinnamic acid); (4) Unknown 2; (5) Caffeoylquinic acid isomer 2 (Hydroxy cinnamic acid); (6) Procyanidin dimer (Anthocyanin); (7) Epicatechin (Flavan‐3‐ol); (8) *p*‐Coumaroylquinic acid isomer 2 (Hydroxy cinnamic acid); (9) Apigenin 6,8‐di‐C‐glucoside (Flavone glycoside); (10) Procyanidin trimer (Flavan‐3‐ol); (11) Procyanidin tetramer (Flavan‐3‐ol); (12) Quercetin‐di‐hexoside (Flavonol); (13) Isorhamnetin diglucoside (Flavonol glycoside); (14) Unknown 9; (15) Unknown 3; (16) Unknown 4; (17) Quercetin‐3‐O‐glucuronide (Flavonol glycoside); (18) Quercetin‐O‐hexoside (Flavonol glycoside); (19) Quercetin‐O‐hexoside (Flavonol glycoside); (20) Quercetin acetyl hexoside (Flavonol glycoside); (21) Cyanidin‐3‐O‐hexoside (Anthocyanin); (22) Unknown 11; (23) Quercetin‐O‐hexoside (Flavonol glycoside); (24) Kaempferol‐O‐acetylhexoside (Flavonol glycoside); (25) Quercetin‐3‐O‐glucoside (Flavonol glycoside); (26) Unknown 12; (27) Unknown 5; (28) Unknown 6; and (29) Quercetin (Flavonol glycoside).

The HPLC‐UV–visible/MS analysis of the methanolic extracts of the three plants is shown in Figure 4. The HPLC‐UV profile permitted to detect the major phenolic compounds while the MS and MS/MS analyses identified them preliminarily. Thus, epicatechin and procyanidin B2 were fully identified (Table 1) according to a commercial standard while the other compounds were identified preliminarily, following UV–Visible, MS, and MS/MS data found in literature. The identification varied from one plant to the other. Thirteen compounds absorbing UV at 280 nm were detected in the *M. oleifera* leaves, among which nine were identified in literature as two hydroxycinnamic acids (caffeoylquinic acid isomers 1 and 2, and *p‐*coumaroylquinic acid isomers 1 and 2), a flavone glycoside (apigenin 6,8‐di‐C‐glucoside), three flavonol glycosides (quercetin‐O‐hexoside, quercetin acetyl‐hexoside, and kaempferol‐O‐acetyl‐hexoside), and an anthocyanin (cyanidin‐3‐O‐hexoside). Quercetin‐O‐hexoside was found as the major phenolic component accounting for 2347 mg HE/kg of powder in this plant. Many studies have shown that the flavonoid glycosides of *M. oleifera* leaves include isoquercetin, kaempferol‐3‐O‐glucoside, apigenin‐8‐C‐glucoside, quercetin 3‐O‐(6′‐O‐malonyl)‐glucoside, and kaempferol 3‐O‐(6′‐O‐malonyl)‐glucoside (Fombang et al., 2021; Karthivashan et al., 2013; Kashiwada et al., 2011; Nouman et al., 2016).

**TABLE 1:** LC‐UV–visible/MS and MS/MS identification and quantification of the main simple phenolic compounds in *Moringa oleifera* leaves, *Xylopia aethiopica* fruits, and *Allium cepa* leaves.

Plant	Peak n°	RT (min)	*λ* max (nm)	[M‐H]^−^ m/z	MS2 m/z (%base peak)	Proposed compound	Content (per kg of powder)
*Moringa Oleifera* leaves	1	8.93	271	570	374 (100), 328 (55), 259 (44), 391 (41)	Unknown 1	
	2	14.62	324	353	191 (100), 179 (60)	Caffeoylquinic acid isomer 1^a^	2020 mg CqAE
	3	19.03	310	337	163 (100), 119 (6)	*p‐c*oumaroylquinic acid isomer 1^b^	269 mg CqAE
	4	22.08	324	367	193 (100), 134 (6)	Unknown 2	
	5	22.47	326	353	179 (100), 173 (87), 191 (9)	Caffeoyl quinic acid isomer 2^a^	805 mg CqAE
	8	28.4	311	337	173 (100), 163 (14), 191 (4)	*p*‐coumaroylquinic acid isomer 2^a^	89 mg CqAE
	9	30.47	270/335	593	473 (100), 503 (31), 353 (29)	Apigenin 6,8‐di‐C‐glucoside^b^ (HPLC‐DAD‐MS/MS profiling)	469 mg HE
	15	39.3	268/336	431	311 (100), 341 (7)	Unknown 3	
	16	39.72	265/347	609	301 (100), 300 (61), 343 (10)	Unknown 4	
	19	40.5	255/353	463	301 (100), 300 (53), 179 (2)	Quercetin‐O‐hexoside^b^ (HPLC‐DAD‐MS/MS profiling; Brito et al., 2014; Goufo et al., 2020; Gouveia & Castilho, 2010)	2347 mg HE
	20	41.88	255/353	505	301 (100), 300 (83), 463 (40)	Quercetin acetyl hexoside^a^ (Goufo et al., 2020)	2187 mg HE
	21	42.58	265/448	447	284 (100), 285 (69), 327 (13)	Cyanidin‐3‐O‐hexoside^b^ (Goufo et al., 2020)	447 mg IE
	24	44.18	265/347	489	285 (100), 284 (14), 286 (3)	Kaempferol‐O‐ acetylhexoside^b^ (Gouveia & Castilho, 2010)	375 mg HE
*Xylopia aethiopica* fruits	2	14.5	325	353	191 (100), 179 (75), 135 (15)	Caffeoylquinic acid isomer 1^a^	95 mg CqAE
	3	18.9	310	337	163 (100), 119 (7)	*p*‐coumaroylquinic acid isomer 1^b^	208 mg CqAE
	5	22.3	326	353	173 (100), 179 (94), 191 (20), 135 (12)	Caffeoyl quinic acid isomer 2^a^	57 mg CqAE
	6	25.32	277	577	425 (100), 407 (53), 451 (21)	Procyanidin dimer B2^b^	189 mg PDE
	7	27.45	278	289	245 (100), 205 (39), 179 (12)	(−)‐Epicatechin^a^	138 mg EE
	8	28.28	311	337	173 (100), 163 (9), 179 (9)	*p‐*coumaroylquinic acid isomer 2^a^	101 mg CqAE
	10	33.16	282/311	865	695 (100), 577 (50), 739 (45)	Procyanidin trimer^b^	146 mg EE
	11	35.7	279	1153	289 (100), 358 (51)	Procyanidin tetramer^b^	57 mg EE
	14	38.3	277	595	300 (100), 455 (17), 463 (16), 301 (11)	Unknown 9	
	17	40.58	350	477	301 (100), 179 (2)	Quercetin‐3‐O‐glucuronide^b^ (Goufo et al., 2020)	61 mg HE
	22	43	285/319	298	298 (100), 135 (46), 299 (17)	Unknown 11	
	26	45.4	270	593	456 (100), 593 (31), 430 (22)	Unknown 12	
	27	46.87	293/317	312	312 (100), 297 (22), 178 (19), 313 (18)	Unknown 5	
*Allium cepa* leaves	12	36.15	344	625	463 (100), 301 (6)	Quercetin di hexoside^b^ (HPLC‐DAD‐MS/MS profiling; Brito et al., 2014)	427 mg HE
	13	37.6	322	639	315 (100), 477 (58), 313 (28), 476 (31)	Isorhamnetin‐di‐hexoside^b^ (Brito et al., 2014)	134 mg HE
	18	40.55	350	463	301 (100), 300 (53), 179 (6)	Quercetin‐O‐hexoside^b^ (HPLC‐DAD‐MS/MS profiling; Brito et al., 2014; Goufo et al., 2020; Gouveia & Castilho, 2010)	64 mg HE
	23	43.3	252/365	463	301 (100)	Quercetin‐O‐hexoside^b^ (HPLC‐DAD‐MS/MS profiling; Brito et al., 2014; Goufo et al., 2020; Gouveia & Castilho, 2010)	1227 mg HE
	25	44.47	290	477	315 (100), 314 (44), 316 (12)	Quercetin‐3‐O‐glucoside^b^ (HPLC‐DAD‐MS/MS profiling; Brito et al., 2014; Goufo et al., 2020; Gouveia & Castilho, 2010)	26 mg HE

	26	46.87	290/314	312	312 (100), 178 (24), 297 (24)	Unknown 5	
	28	47.45	289/318	342	342 (100), 327 (87), 343 (24)	Unknown 6	
29	48.55	350	301	179 (100), 151 (53), 301 (37)	Quercetin^a^	191 mg HE

Abbreviations: CqAE, 5‐Caffeoylquinic Acid; EE, Epicatechin equivalent; HE, hyperoside equivalent; IE, ideain equivalent; PDE: Procyanidin dimer B2; RT, retention time; [M‐H]^−^, Deprotonated molecule; *λ* max, wavelengths of maximum absorption.

^a^ Identified according to a commercial standard or standard purified in the laboratory.

^b^ Identified according to the m/z and UV–visible data found in literature.

Thirteen phenolic phytochemicals were detected in the *X. aethiopica* fruits among which nine compounds identified were hydroxycinnamic acids (caffeoylquinic acid isomers 1 and 2, and *p‐*coumaroylquinic acid isomers 1 and 2), flavan‐3‐ols (procyanidin dimer B2, (−)‐epicatechin, procyanidin trimer, and procyanidin tetramer), and flavonol glycoside (quercetin‐3‐O‐glucuronide). The most abundant compound was *p‐*coumaroylquinic acid isomer 1 (208 mg CqAE/kg of powder). Previous research had shown that gallic acid, chlorogenic acid, caffeic acid, T‐cinnamic acid, catechin, epicatechin, and eugenol were present in *X. aethiopica* (Sokamte et al., 2019). A recent study conducted by Oguntona et al. (2022) detected 87 compounds in *Xylopia aethiopica* leaves by GC–MS with their relative percentage taking into consideration the sum of all eluted peaks as 100%. The major compounds identified were the andrographolide, bicyclo[3.1.0]hexan‐2‐ol, 2methyl‐5‐(1‐methylethyl), 1H‐naphthol[2,1b]pyran ethenyldodecahydro3,4a,7,7,10a‐pentamethyl‐,[3R‐(3,alpha, 4a,beta, 6a alpha, 10a beta, and 10b alpha)], (1S)2,6,6‐trimethylbicyclo[3.1.1]hept‐2‐ene, and beta‐copaene. The differences in the phenolic profiles may be due to the state of physiological maturity of the plant, the part of plant, and the type of soil (Du Toit et al., 2020; Matinez‐Ramos et al., 2020).

In the *A. cepa* leaves, eight phenolic compounds were found, five of which were identified as flavonol glycosides (quercetin dihexoside, isorhamnetin‐dihexoside, quercetin‐O‐hexoside, and quercetin‐3‐O‐glucoside) and a flavonol (quercetin). A quercetin‐O‐hexoside was identified as the major phenolic compound accounting for 1227 mg HE per kg of dry powder of this plant. Similarly, Tedesco et al. (2015) reported that quercetin was the main compound present in *A. cepa*, accounting for about 80%–95% of the total flavonol content. Recent research showed in *A. cepa* the presence of protocatechuic acid, *p*‐hydroxybenzoic acid, vanillinic acid, *p*‐cumaric acid, quercetin, quercetin‐3‐O‐glucoside, quercetin‐4‐O‐glucoside, myricetin, kaempferol, isorhamnetin, quercetin‐3‐rhamnoside, and isorhamnetin‐3‐glucoside (Óscar et al., 2020). The variability of phenolic compounds in plants can be due not only to differences in the extraction solvents and analytical techniques used but also to the plant environment, climate, season, plant age, plant type, plant genetic program, cultural practices, maturity, and/or soil type (Matinez‐Ramos et al., 2020; Mykhailenko et al., 2020).

The groups of secondary metabolites (hydroxycinnamic acid, flavone, anthocyanin, flavan‐3‐ol, and flavonol) detected in the plant parts could justify the radical scavenging and ferric reducing powers observed previously in their different extracts. Of these, quercetin and its glycosides, detected mainly in *A. cepa* and *M. oleifera* have longer retention times than the other compounds. This could rather be due to its polarity due to the hydrogen bonding which could otherwise lead to an increase in the boiling temperature. The hydrophobicity of these molecules makes them more soluble in lipids where they scavenge free radicals and bind transition metal ions. These properties of quercetin enable it to inhibit lipid peroxidation (Azizi et al., 2019; Parcheta et al., 2021).

### 3.4 Effect of plant extracts on fatty acid composition of fish during processing

Changes in the fatty acid composition of oils extracted from smoked and sun‐dried fish are presented in Tables 2 and 3, respectively. These processes significantly reduced the presence of PUFAs and omega‐3 fatty acids in the fish (*p* < .05). Eicosapentaenoic acid (C20:5n‐3, EPA) and docosahexaenoic acid (C22:6n‐3, DHA) were the most affected. The percentage of loss of total omega‐3 fatty acids was more than 85%. This decrease in the PUFA content of the lipids during the processing is potentially attributed to the structural and chemical changes induced in fish cells during exposure to sunshine and smoke. The sun and high temperatures facilitate the attack of the double bonds of the unsaturated fatty acids, resulting in lipid oxidation and a decrease in the nutritive value of fish oil (Khaoula et al., 2013). During smoking, AHE (12 g/L), MAE (40 g/L), and AAE (40 g/L) significantly protected the omega‐3 fatty acids (*p* < .05), when compared to fish processed with water only (Table 2). On the other hand, all the extracts at the different concentrations significantly protected the omega‐3 fatty acids during the sun‐drying process (*p* < 0.05), when compared to fish processed with water only (Table 3). At 40 g/L, AAE protected more omega‐3 fatty acids than all the extracts, including BHT at 0.2 g/L, during the two processes. Similar results were obtained by Chaula et al. (2019) who demonstrated that the aqueous extracts of *Syzygium aromaticum* and *Kappaphycus alvarezii* protected fish (*Rastrineobola argentea*) lipids against oxidation during sun drying. Messina et al. (2019, 2021) also showed that cold smoking combined with antioxidants had a positive effect on lipid peroxidation of meager (*Argyrosomus regius*) fillets, lower values of malondialdehyde, and protected omega‐3 in fish during the process. Moreover, as in the case of *Sander lucioperca* filets, a fatty fish species, the lipid oxidation results showed that the combined application of *Dunaliella salina* as natural antioxidant and smoking significantly reduced the oxidation in *Sander lucioperca* in comparison with the batch that was only smoked (Bouriga et al., 2022).

**TABLE 2:** Changes in fatty acid composition of lipids extracted from smoked fish (%FA/TFA).

Fatty acid	FF	Control	F + BHT 0.2 g/L	F + MHE 12 g/L	F + XHE 12 g/L	F + AHE 12 g/L	F + MAE 40 g/L	F + XAE 40 g/L	F + AAE 40 g/L
C14:0	5.76 ± 0.01^a^	1.39 ± 0.01^h^	2.49 ± 0.01^e^	3.30 ± 0.00^b^	1.90 ± 0.00^f^	2.65 ± 0.07^d^	1.50 ± 0.00^g^	0.70 ± 0.00^i^	3.00 ± 0.00^c^
C15:0	0.96 ± 0.00^a^	0.25 ± 0.07^de^	0.60 ± 0.00^c^	0.20 ± 0.00^e^	0.20 ± 0.00^e^	0.80 ± 0.00^b^	0.30 ± 0.00^d^	0.20 ± 0.00^e^	0.80 ± 0.00^b^
C16:0	27.68 ± 0.01^a^	22.15 ± 0.07^e^	23.10 ± 0.00^c^	22.55 ± 0.07^d^	20.85 ± 0.07^f^	24.85 ± 0.07^b^	19.80 ± 0.00^h^	20.45 ± 0.07^g^	18.80 ± 0.14^i^
C18:0	5.62 ± 0.01^i^	8.50 ± 0.00^d^	9.20 ± 0.00^b^	8.20 ± 0.00^e^	8.00 ± 0.00^f^	8.85 ± 0.07^c^	7.20 ± 0.00^g^	9.50 ± 0.00^a^	7.05 ± 0.07^h^
C20:0	0.20 ± 0.00^c^	0.30 ± 0.00^ab^	0.25 ± 0.07^bc^	0.20 ± 0.00^c^	0.20 ± 0.00^c^	0.30 ± 0.00^ab^	0.20 ± 0.00^c^	0.35 ± 0.07^a^	0.30 ± 0.00^ab^
∑SFA	40.21 ± 0.01^a^	32.59 ± 0.00^e^	35.64 ± 0.08^c^	34.45 ± 0.07^d^	31.15 ± 0.07^f^	37.45 ± 0.21^b^	29.00 ± 0.00^h^	31.20 ± 0.00^f^	29.95 ± 0.21^g^
C16:1(n‐9)	8.53 ± 0.03^c^	4.45 ± 0.07^g^	8.80 ± 0.00^b^	6.00 ± 0.00^e^	4.90 ± 0.00^f^	6.85 ± 0.07^d^	5.00 ± 0.00^f^	3.50 ± 0.00^h^	9.85 ± 0.07^a^
C18:1(n‐9)	16.47 ± 0.01^i^	42.65 ± 0.21^d^	35.90 ± 0.00^f^	42.35 ± 0.07^e^	44.75 ± 0.07^b^	31.75 ± 0.07^h^	43.65 ± 0.21^c^	45.05 ± 0.07^a^	32.65 ± 0.07^g^
C20:1	0.54 ± 0.01^e^	1.10 ± 0.00^cd^	1.40 ± 0.00^ab^	1.00 ± 0.00^d^	1.30 ± 0.00^abc^	1.25 ± 0.07^abc^	1.25 ± 0.07^abc^	1.45 ± 0.21^a^	1.20 ± 0.00^bc^
∑MUFA	25.53 ± 0.03^h^	48.20 ± 0.14^d^	44.10 ± 0.00^e^	49.35 ± 0.07^c^	50.95 ± 0.07^a^	39.85 ± 0.21^g^	49.90 ± 0.14^b^	50.10 ± 0.14^b^	43.70 ± 0.00^f^
C20:2	0.26 ± 0.00^**e**^	0.70 ± 0.00^**a**^	0.50 ± 0.14^**bcd**^	0.50 ± 0.00^**bcd**^	0.45 ± 0.07^**cd**^	0.55 ± 0.07^**abc**^	0.50 ± 0.00^**bcd**^	0.65 ± 0.07^**ab**^	0.35 ± 0.07^**de**^
C18:3(n‐3)	3.95 ± 0.01^a^	0.70 ± 0.00^e^	2.10 ± 0.00^c^	0.70 ± 0.00^e^	0.75 ± 0.07^e^	2.10 ± 0.00^c^	1.30 ± 0.00^d^	0.00 ± 0.00^f^	3.65 ± 0.07^b^
C20:3(n‐3)	0.66 ± 0.00^a^	0.00 ± 0.00^e^	0.40 ± 0.00^c^	0.00 ± 0.00^e^	0.00 ± 0.00^e^	0.35 ± 0.07^c^	0.20 ± 0.00^d^	0.20 ± 0.00^d^	0.50 ± 0.00^b^
C20:5(n‐3)	2.34 ± 0.01^a^	0.00 ± 0.00^d^	1.00 ± 0.00^c^	0.00 ± 0.00^d^	0.00 ± 0.00^d^	1.00 ± 0.00^c^	0.00 ± 0.00^d^	0.00 ± 0.00^d^	2.00 ± 0.00^b^
C22:6(n‐3)	9.72 ± 0.01^a^	1.50 ± 0.71^bc^	2.00 ± 0.00^b^	1.00 ± 0.00^c^	1.00 ± 0.00^c^	2.00 ± 0.00^b^	1.00 ± 0.00^c^	1.50 ± 0.71^bc^	2.00 ± 0.00^b^
C18:2(n‐6)	3.86 ± 0.01^h^	11.10 ± 0.14^b^	8.20 ± 0.00^f^	10.35 ± 0.07^d^	11.25 ± 0.07^b^	9.80 ± 0.00^e^	11.65 ± 0.07^a^	10.75 ± 0.07^c^	7.85 ± 0.07^g^
C18:3(n‐6)	0.61 ± 0.00^a^	0.30 ± 0.00^c^	0.20 ± 0.00^d^	0.30 ± 0.00^c^	0.40 ± 0.00^b^	0.30 ± 0.00^c^	0.20 ± 0.00^d^	0.65 ± 0.07^a^	0.35 ± 0.07^bc^
C20:3(n‐6)	0.55 ± 0.00^ef^	0.75 ± 0.07^ab^	0.65 ± 0.07^cd^	0.50 ± 0.00^f^	0.60 ± 0.00^de^	0.70 ± 0.00^bc^	0.80 ± 0.00^a^	0.70 ± 0.00^bc^	0.60 ± 0.00^de^
C20:4(n‐6)	1.26 ± 0.01^g^	1.40 ± 0.00^ef^	2.00 ± 0.14^c^	1.10 ± 0.00^h^	1.30 ± 0.00^fg^	2.20 ± 0.00^b^	1.70 ± 0.00^d^	1.45 ± 0.07^e^	3.15 ± 0.07^a^
∑PUFA	23.19 ± 0.03^a^	16.30 ± 0.42^e^	16.40 ± 0.00^e^	14.25 ± 0.07^f^	15.85 ± 0.07^e^	18.90 ± 0.42^c^	18.10 ± 0.00^d^	16.00 ± 0.00^e^	20.75 ± 0.21^b^
∑n‐3	16.66 ± 0.02^a^	2.40 ± 0.14^f^	4.85 ± 0.07^d^	1.50 ± 0.00^h^	1.85 ± 0.07^g^	5.35 ± 0.35^c^	3.25 ± 0.07^e^	1.80 ± 0.00^gh^	8.45 ± 0.07^b^
∑n‐6	6.28 ± 0.01^g^	13.55 ± 0.07^b^	11.05 ± 0.21^f^	12.25 ± 0.07^d^	13.55 ± 0.07^b^	13.00 ± 0.00^c^	14.35 ± 0.07^a^	13.55 ± 0.07^b^	11.95 ± 0.07^e^
n‐3/n‐6	2.65 ± 0.00^a^	0.18 ± 0.01^f^	0.44 ± 0.01^c^	0.12 ± 0.00^g^	0.14 ± 0.01^g^	0.41 ± 0.03^d^	0.23 ± 0.01^e^	0.13 ± 0.00^g^	0.71 ± 0.00^b^
PUFA/SFA	0.58 ± 0.00^c^	0.50 ± 0.01^d^	0.46 ± 0.00^e^	0.41 ± 0.00^f^	0.51 ± 0.00^d^	0.50 ± 0.01^d^	0.62 ± 0.00^b^	0.51 ± 0.00^d^	0.69 ± 0.01^a^

*Note*: Mean values in the same column with different superscript letters are significantly different (*p* < .05).

Abbreviations: BHT, butylated hydroxytoluene; Control, fish without extract and BHT; FA, fatty acid; FF, fresh fish; F + BHT0.2 g/L, fish treated with BHT at 0.2 g/L concentration; F + MHE, fish treated with *M. oleifera* hydroethanolic extracts; F + MAE, fish treated with *M. oleifera* aqueous extracts; F + XHE, fish treated with *X. aethiopica* hydroethanolic extracts; F + XAE, fish treated with *X. aethiopica* aqueous extracts; F + AHE, fish treated with *A. cepa* hydroethanolic extracts; F + AAE, fish treated with *A. cepa* aqueous extracts; 12 g/L, concentration of hydroethanolic extracts; 40 g/L, concentration of aqueous extracts; MUFA, monounsaturated fatty acid; PUFA, polyunsaturated fatty acid; SFA, saturated fatty acid; TFA, total fatty acid.

**TABLE 3:** Changes in fatty acid composition of lipids extracted from sun‐dried fish (%FA/TFA).

Fatty acid	FF	Control	F + BHT 0.2 g/L	F + MHE 12 g/L	F + XHE 12 g/L	F + AHE 12 g/L	F + MAE 40 g/L	F + XAE 40 g/L	F + AAE 40 g/L
C14:0	5.76 ± 0.01^a^	3.05 ± 0.01^d^	3.20 ± 0.00^c^	2.80 ± 0.00^f^	2.90 ± 0.00^e^	1.90 ± 0.00^g^	3.05 ± 0.07^d^	1.80 ± 0.00^h^	3.75 ± 0.07^b^
C15:0	0.96 ± 0.00^b^	0.20 ± 0.00^d^	0.50 ± 0.00^c^	0.55 ± 0.07^c^	0.50 ± 0.00^c^	0.45 ± 0.07^c^	0.50 ± 0.00^c^	0.50 ± 0.00^c^	1.25 ± 0.07^a^
C16:0	27.68 ± 0.01^a^	21.10 ± 0.00^g^	22.00 ± 0.00^e^	21.00 ± 0.00^g^	22.20 ± 0.00^d^	21.55 ± 0.07^f^	20.60 ± 0.00^h^	23.90 ± 0.00^b^	23.30 ± 0.14^c^
C18:0	5.62 ± 0.01^h^	7.20 ± 0.00^g^	8.45 ± 0.07^c^	7.70 ± 0.00^d^	8.80 ± 0.00^b^	7.60 ± 0.00^e^	7.40 ± 0.00^f^	8.40 ± 0.00^c^	9.60 ± 0.00^a^
C20:0	0.20 ± 0.00^c^	0.20 ± 0.00^c^	0.40 ± 0.00^a^	0.30 ± 0.00^b^	0.25 ± 0.07^bc^	0.20 ± 0.00^c^	0.30 ± 0.00^b^	0.30 ± 0.00^b^	0.40 ± 0.00^a^
∑SFA	40.21 ± 0.01^a^	31.75 ± 0.01^f^	34.55 ± 0.07^d^	32.35 ± 0.07^e^	34.65 ± 0.07^d^	31.70 ± 0.00^f^	31.85 ± 0.07^f^	34.90 ± 0.00^c^	38.30 ± 0.14^b^
C16:1(n‐9)	8.53 ± 0.03^b^	6.30 ± 0.00^de^	6.50 ± 0.14^d^	7.80 ± 0.14^c^	6.10 ± 0.00^ef^	5.45 ± 0.07^g^	8.50 ± 0.28^b^	5.80 ± 0.00^fg^	10.40 ± 0.28^a^
C18:1(n‐9)	16.47 ± 0.01^i^	43.80 ± 0.00^a^	39.30 ± 0.14^d^	38.75 ± 0.07^e^	38.10 ± 0.00^f^	41.20 ± 0.14^b^	35.00 ± 0.14^g^	39.60 ± 0.14^c^	25.15 ± 0.07^h^
C20:1	0.54 ± 0.01^c^	0.90 ± 0.00^b^	1.00 ± 0.28^b^	0.95 ± 0.21^b^	1.10 ± 0.00^b^	0.85 ± 0.07^b^	1.55 ± 0.07^a^	0.95 ± 0.07^b^	1.10 ± 0.00^b^
∑MUFA	25.53 ± 0.03^h^	51.00 ± 0.00^a^	46.80 ± 0.00^c^	47.50 ± 0.00^b^	45.30 ± 0.00^e^	47.50 ± 0.14^b^	45.05 ± 0.07^f^	46.35 ± 0.07^d^	36.65 ± 0.21^g^
C20:2	0.26 ± 0.00^c^	0.30 ± 0.00^bc^	0.50 ± 0.14^a^	0.35 ± 0.07^bc^	0.40 ± 0.00^ab^	0.40 ± 0.00^ab^	0.30 ± 0.00^bc^	0.40 ± 0.00^ab^	0.30 ± 0.00^bc^
C18:3(n‐3)	3.95 ± 0.01^a^	0.90 ± 0.00^g^	1.30 ± 0.00^e^	1.95 ± 0.07^d^	1.15 ± 0.07^f^	1.35 ± 0.07^e^	2.40 ± 0.00^c^	1.30 ± 0.00^e^	3.15 ± 0.07^b^
C20:3(n‐3)	0.66 ± 0.00^a^	0.10 ± 0.00^e^	0.20 ± 0.00^d^	0.30 ± 0.00^c^	0.20 ± 0.00^d^	0.20 ± 0.00^d^	0.30 ± 0.00^c^	0.20 ± 0.00^d^	0.50 ± 0.00^b^
C20:5(n‐3)	2.34 ± 0.01^a^	0.30 ± 0.00^f^	0.35 ± 0.07^ef^	0.80 ± 0.00^d^	0.45 ± 0.07^e^	0.35 ± 0.07^ef^	1.00 ± 0.00^c^	0.45 ± 0.07^e^	1.65 ± 0.07^b^
C22:6(n‐3)	9.72 ± 0.01^a^	0.55 ± 0.07^h^	1.80 ± 0.00^e^	2.00 ± 0.00^d^	2.40 ± 0.00^c^	1.30 ± 0.00^g^	1.75 ± 0.07^e^	1.50 ± 0.00^f^	3.80 ± 0.00^b^
C18:2(n‐6)	3.86 ± 0.01^i^	11.30 ± 0.00^b^	8.60 ± 0.00^f^	8.00 ± 0.00^g^	9.60 ± 0.00^d^	11.55 ± 0.07^a^	9.75 ± 0.07^c^	9.35 ± 0.07^e^	6.00 ± 0.00^h^
C18:3(n‐6)	0.61 ± 0.00^a^	0.30 ± 0.00^bc^	0.20 ± 0.00^c^	0.20 ± 0.00^c^	0.35 ± 0.07^b^	0.20 ± 0.00^c^	0.25 ± 0.07^bc^	0.20 ± 0.00^c^	0.25 ± 0.07^bc^
C20:3(n‐6)	0.55 ± 0.00^c^	0.55 ± 0.07^c^	0.70 ± 0.00^a^	0.60 ± 0.00^bc^	0.60 ± 0.00^bc^	0.70 ± 0.00^a^	0.60 ± 0.00^bc^	0.65 ± 0.07^ab^	0.60 ± 0.00^bc^
C20:4(n‐6)	1.26 ± 0.01^f^	1.00 ± 0.00^g^	1.80 ± 0.00^d^	2.00 ± 0.00^c^	2.00 ± 0.00^c^	1.50 ± 0.00^e^	2.20 ± 0.00^b^	1.60 ± 0.00^e^	3.30 ± 0.14^a^
∑PUFA	23.19 ± 0.03^a^	15.30 ± 0.00^g^	15.45 ± 0.21^g^	16.20 ± 0.00^f^	17.15 ± 0.21^de^	17.55 ± 0.07^d^	18.55 ± 0.07^c^	15.65 ± 0.21^g^	19,55 ± 0.21^b^
∑n‐3	16.66 ± 0.02^a^	**1.85 ± 0.07** ^**h**^	3.65 ± 0.07^f^	5.05 ± 0.07^d^	4.20 ± 0.14^e^	3.20 ± 0.14^g^	5.45 ± 0.07^c^	3.45 ± 0.07^f^	9.10 ± 0.14^b^
∑n‐6	6.28 ± 0.01^i^	13.15 ± 0.07^b^	11.30 ± 0.00^f^	10.80 ± 0.00^g^	12.55 ± 0.07^d^	13.95 ± 0.07^a^	12.80 ± 0.00^c^	11.80 ± 0.14^e^	10.15 ± 0.07^h^
n‐3/n‐6	2.65 ± 0.00^a^	0.14 ± 0.01^h^	0.32 ± 0.01^e^	0.47 ± 0.01^c^	0.33 ± 0.01^e^	0.23 ± 0.01^g^	0.43 ± 0.01^d^	0.29 ± 0.00^f^	0.90 ± 0.01^b^
PUFA/SFA	0.58 ± 0.00^a^	0.48 ± 0.00^e^	0.45 ± 0.01^f^	0.50 ± 0.00^cd^	0.49 ± 0.01^d^	0.55 ± 0.00^b^	0.58 ± 0.00^a^	0.45 ± 0.01^f^	0.51 ± 0.00^c^

*Note*: Mean values in the same column with different superscript letters are significantly different (*p* < .05).

Abbreviations: BHT, butylated hydroxytoluene; Control, fish without extract and BHT; FA, fatty acid; FF, fresh fish; F + BHT0.2 g/L, fish treated with BHT at 0.2 g/L concentration; F + MHE, fish treated with *M. oleifera* hydroethanolic extracts; F + MAE, fish treated with *M. oleifera* aqueous extracts; F + XHE, fish treated with *X. aethiopica* hydroethanolic extracts; F + XAE, fish treated with *X. aethiopica* aqueous extracts; F + AHE, fish treated with *A. cepa* hydroethanolic extracts; F + AAE, fish treated with *A. cepa* aqueous extracts; 12 g/L, concentration of hydroethanolic extracts; 40 g/L, concentration of aqueous extracts; MUFA, monounsaturated fatty acid; PUFA, polyunsaturated fatty acid; SFA, saturated fatty acid; TFA, total fatty acid.

This activity can be attributed to the polyphenolic compounds in these plant extracts (Figures 1 and 3). The hydrophobicity of quercetins (detected by HLPC mainly in the *A. cepa* leaves) makes them more soluble in lipids than other phenolic compounds. Indeed, quercetin and its derivatives scavenge free radicals and bind transition metal ions (Parcheta et al., 2021). This may justify why at a concentration of 40 g/L, the AAE provided better prevention of the oxidation of omega‐3 fatty acids of fish during processing than the extracts of the other two plants and BHT at 0.2 g/L. According to Layé et al. (2015), the omega‐3 fatty acids present in this fish (such as EPA and DHA) have preventive effects on human coronary artery and Alzheimer's disease. Previous research demonstrated that DHA is essential for the development of the fetal brain and the eye retina (San & Chew, 2005).

### 3.5 Principal component analysis of phenolic compounds and antioxidant activity

**FIGURE 5:**
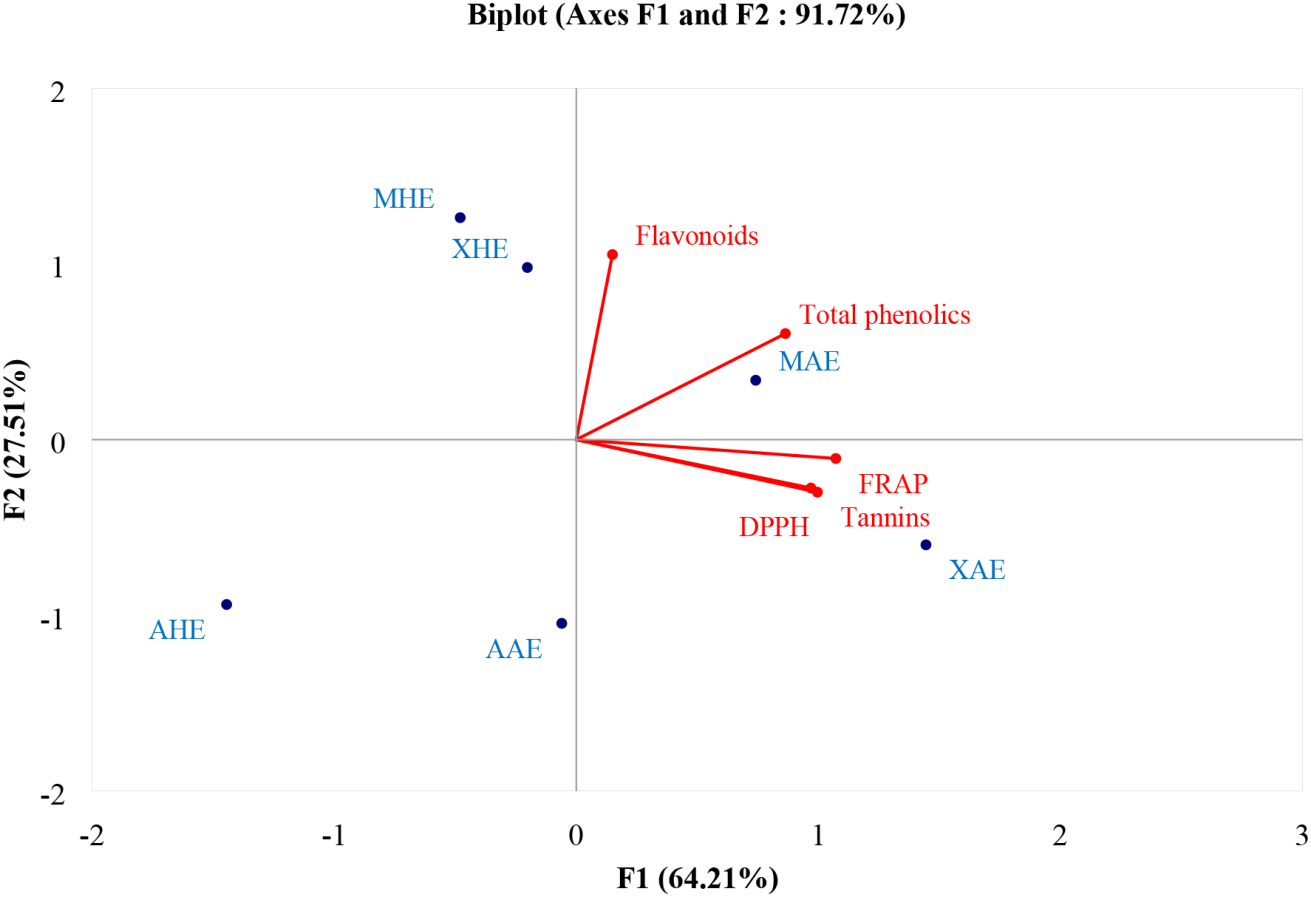
Principal Components of phenolic compounds and antioxidant data. AAE, *A. cepa* Aqueous Extracts; AHE, *A. cepa* Hydroethanolic Extracts; MAE, *M. oleifera* AqueousExtracts; MHE, *M. oleifera* Hydroethanolic Extracts; XAE, *X. aethiopica* Aqueous Extracts; XHE, *X. aethiopica* Hydroethanolic Extracts.

Figure 5 shows the biplot of the principal component analysis (PCA) of the phenolic compounds and the antioxidant activities of the plant extracts. Two components proved to be more interesting for the analysis: Principal Component 1 (F1) and Principal Component 2 (F2). They represented 91.72% of the initial variables, with 64.21% and 27.51% for the F1 and F2 axes, respectively. The PCA makes it possible to visualize the distribution of the various plant extracts which are presented in the form of points, according to Principal Components 1 and 2. The vectors representing the phenols, flavonoids, tannins, DPPH, and FRAP are oriented in one direction, pointing to the positive part of F1, while the tannins, FRAP, and DPPH point to the negative part of F2 and the phenols and flavonoids are in its positive part. The vectors are all quite far from 0, and the angles they form are less than 90°C. This suggests that the methods (FRAP and DPPH) correlate with the phenolic compounds which could be responsible for the scavenging of the DPPH^·^ radical and the ferric reducing powers observed previously.

The strong positive correlation between the tannins and FRAP (*r* = .88; *p* < .05) and the tannins and DPPH (*r* = .82; *p* < .05) shows that the antioxidant powers of the extracts seem to be more related to the presence of these compounds. Flavonoids are the most abundant of the two groups of phenolic compounds analyzed because they are more correlated with the total phenols (*r* = .57; *p* < .05). This observation correlates with the results of the phenol composition. According to Francesco and Tory (2007), flavonoids are the most abundant phenolic compounds in the plant. Figure 4 suggests that MAE is richer in phenol content while XAE is richer in tannins and has better antioxidant power.

## 4 CONCLUSION

The results of the present investigation showed that the total phenolic, flavonoid, and tannin contents varied from one plant extract to the other. The plants contained hydroxycinnamic acid, anthocyanin, flavone glycoside, flavan‐3‐ol, and flavonol. The radical scavenging and ferric‐reducing antioxidant powers increased with the concentration of the plant extracts which protected the fish lipids from oxidative damage. The aqueous extract of *A. cepa* at 40 g/L best preserved the omega‐3 fish lipids during smoking and sun drying.

*M. oleifera* leaf, *X. aethiopica* fruit, and *A. cepa* leaf extracts can therefore be used to prevent fatty acid oxidation in fish lipids during smoking and sun drying. These plants can be exploited as alternative sources of antioxidants to prevent fatty acid oxidation in all oil systems.

Future research must be focused on determining the nutritional composition as well as the protein quality of fish treated with plant extracts and then smoked or sun dried.

## AUTHOR CONTRIBUTIONS

**Roger Ponka:** Conceptualization (lead); investigation (lead); methodology (lead); project administration (lead); supervision (lead); validation (lead); visualization (lead); writing – review and editing (lead). **Sylvain Guyot:** Data curation (equal); investigation (equal); methodology (equal); software (equal); validation (equal); visualization (equal); writing – review and editing (equal). **Anne Meynier:** Data curation (equal); formal analysis (equal); methodology (equal); software (equal); validation (equal); visualization (equal); writing – review and editing (equal). **Germain Kansci:** Conceptualization (equal); data curation (equal); investigation (equal); methodology (equal); software (equal); validation (equal); visualization (equal); writing – review and editing (equal). **Noël Tenyang:** Conceptualization (equal); data curation (equal); formal analysis (equal); investigation (equal); methodology (equal); software (equal); validation (equal); writing – review and editing (equal). **Godefroy Tabanty:** Conceptualization (equal); data curation (equal); formal analysis (equal); investigation (equal); methodology (equal); resources (equal); software (equal); validation (equal); visualization (equal); writing – original draft (lead). **Lucie Birault:** Formal analysis (equal); investigation (equal); methodology (equal); software (equal); visualization (equal); writing – review and editing (equal). **Alice Kermarrec:** Data curation (equal); methodology (equal); software (equal); visualization (equal); writing – review and editing (equal). **Agnes Gacel:** Data curation (equal); formal analysis (equal); methodology (equal); software (equal); writing – review and editing (equal).

## FUNDING INFORMATION

This research did not receive any specific grant from funding agencies in the public, commercial, or not‐for‐profit sectors.

## CONFLICT OF INTEREST STATEMENT

The authors declare no conflict of interest.

## ETHICS STATEMENT

This study does not include any animal or human testing.

## CONSENT TO PARTICIPATE

All the co‐authors participated in the preparation of this manuscript.

## CONSENT FOR PUBLICATION

All authors have read and agreed to the published version of the manuscript. All authors read and approved the final manuscript.

## Data Availability

Data are available on request from the authors.
